# Clinical description and treatment outcomes of *Paederus* dermatitis in Phuentsholing, Bhutan in 2021: A cross‐sectional study

**DOI:** 10.1002/ski2.223

**Published:** 2023-02-23

**Authors:** Kinley Gyeltshen, Ngawang Sangye, Kunzang C. Tenzin, Thinley Dorji

**Affiliations:** ^1^ Inpatient and Outpatient Department Gidakom Hospital Ministry of Health Thimphu Bhutan; ^2^ Inpatient and Outpatient Department Trashigang General Hospital Trashigang Bhutan; ^3^ Emergency Department Phuentsholing Hospital Ministry of Health Phuentsholing Bhutan; ^4^ Department of Internal Medicine Central Regional Referral Hospital Gelegphu Bhutan

## Abstract

An increasing number of beetle population and outbreaks of irritant contact dermatitis are reported from newer geographic locations. Bhutan is one such country that witnessed an outbreak of *Paederus* dermatitis (PD) in Phuentsholing sub‐district in 2021. This study describes the clinical symptoms, skin lesions and treatment outcomes of PD in Bhutan. This was a descriptive cross‐sectional study of an outbreak of *Paederus*‐related contact dermatitis in Phuentsholing, Bhutan. Clinical symptoms, skin lesions, duration of illness, recovery time and response to treatment were recorded. Of the 81 patients with PD, the males constituted 54% (44) and the mean age was 22 years (range: 1–51 years). The commonly affected groups were those aged 11–20 years (40.7%) and school or college students (50.6%). The common symptoms were pain, itching, redness, tenderness and blister formation. The lesions were erythematovesicular (70%), linear (54.3%) and kissing lesions (28.4%). All patients received some form of topical or oral steroid therapy and recovery was 100%. The mean duration from the onset till the recovery of the skin rash was 13 days (SD ± 8.3 days). PD outbreak is a self‐limiting form of contact dermatitis. This is the first report of PD in the sub‐Himalayan region and may be linked to climate change. There is a need for active surveillance and monitoring of such emerging weather/climate‐related agents for appropriate health system response in disease prevention and treatment.

## Graphical abstract

Of the 81 patients with *Paederus* dermatitis, the males constituted 54% (44) and the mean age was 22 years (range: 1–51 years). The commonly affected groups were those aged 11–20 years (40.7%) and school or college students (50.6%). The common symptoms were pain, itching, redness, tenderness and blister formation. The lesions were erythematovescicular (70%), linear (54.3%) and kissing lesions (28.4%). All patients received some form of topical or oral steroid therapy and recovery was 100%. The mean duration from the onset till the recovery of skin rash was 13 days (SD ± 8.3 days).

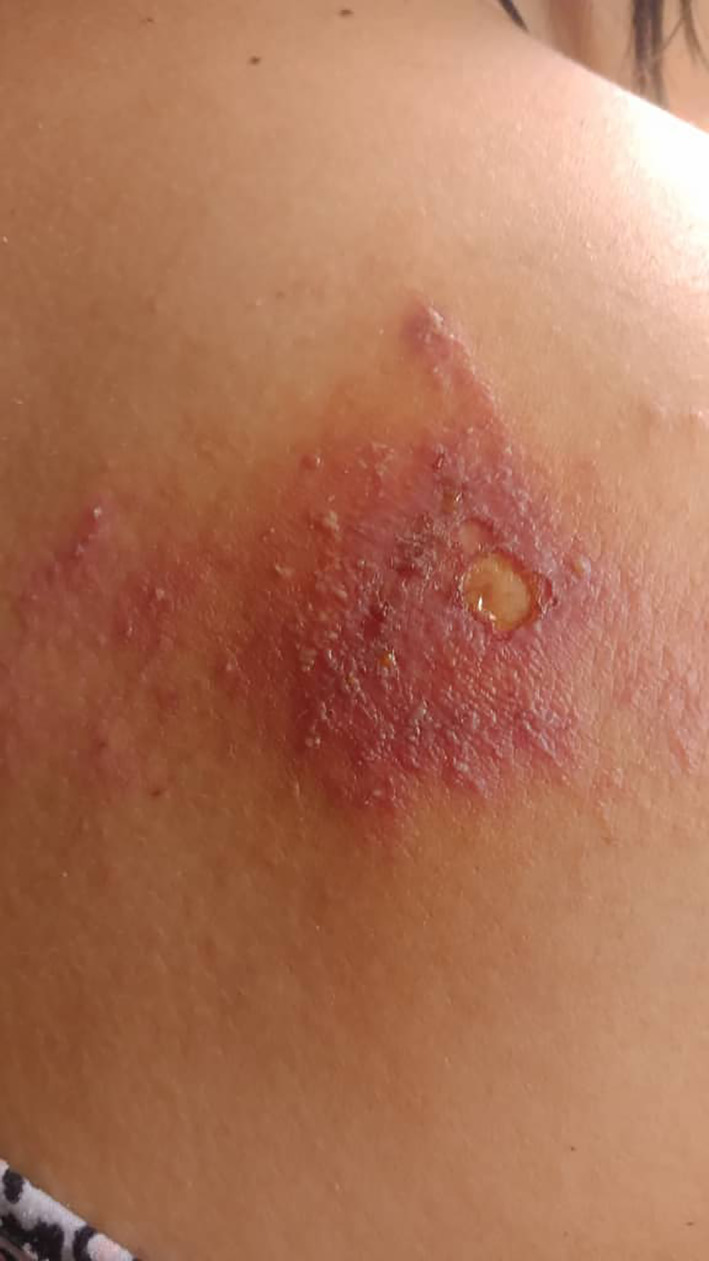

1

**What is already known about this topic?**

- It is known that *Paederus* beetles can cause irritant contact dermatitis.

**What does this study add?**

- This study highlights the occurrence of this condition in newer locations indicating a possible link to climate change. It also gives additional information on common clinical symptoms, signs and treatment of *Paederus* dermatitis.

## 2 BACKGROUND

The health of human populations is sensitive to shifts in weather patterns and a shifting pattern of disease agents which, in part, are linked to ecological disruptions and climate change.^1^ One such example is the spread of beetles into warm and humid places in the tropics leading to disease outbreaks in newer locations. Beetles constitute an order with the highest number of species known and account for roughly 25% of approximately 1.5 million described species.^2^ Of these, about 622 species are the *Paederus* genus belonging to the *Staphyllinadae* family and *Paederinae* subfamily. It is distributed worldwide, except in Antarctica. The size of *Paederus* beetles is around one and a half times that of a mosquito‐usually 7–10 mm long and 0.5 mm wide. They live in moist habitats and feed on small insects and plant debris.^3^, ^4^

Of the described *Paederus* genus, about 30 of them have been shown to cause linear dermatitis or to contain the toxic agent known as paederin. *Paederus* dermatitis (PD) or Blister beetle dermatitis is an irritant contact dermatitis due to the accidental crush of insects belonging to the *Paederus* family on the skin.^5^ This beetle does not bite or sting but accidental crushing or brushing against the skin causes the release of its haemolymph containing paederin that causes painful necrotic blisters. *Paederus* beetles have been associated with outbreaks of dermatitis in various countries across the world including Sri Lanka and India in the South Asia region.^6^ PD is commonly caused by *Paederus melampus* in India and is reported in Odisha, West Bengal, Punjab, Rajasthan and Tamil Nadu.^7^ This condition is commonly seen during or after rainy seasons.

An unusually high number of dermatitis cases were observed in Phuentsholing, Bhutan during the peak summer of 2021 and 2022. The disease was characterized by a painful and itchy skin rash that developed overnight and caused significant discomfort and disruption of daily routine activities. The lesions were characterized by poor response to antibiotics and other conventional treatments such as antihistamines. An outbreak of such skin conditions at place and time during the rainy season suggested a possible aetiological agent related to climate and weather.

In 2021 in Phuentsholing, Bhutan, the first group of victims with unusual dermatitis was recorded among those living in clusters settlements such as the boarding students at schools and colleges. The second group of victims was among workers at the food auction yard and the temporary shelter in Toorsa where hundreds of people resided while only a few cases originated from elsewhere in Phuentsholing. We have recorded more than 100 such cases; of whom the majority of them had exposure to *Paederus* beetles that are found inhabiting their locality. In 2022, another outbreak with more than 300 cases of similar skin conditions was reported^8^ where more than 80% of the victims were exposed to the same beetles. This outbreak of unusual skin rash is new to the local inhabitants. We also studied and discussed the symptoms and signs of dermatitis, duration of illness, treatment and possible preventive methods and their association with exposure to beetles in their locality among patients who presented to the Phuentsholing General Hospital in 2021.

## 3 METHODS

### 3.1 Study design and setting

This was a descriptive cross‐sectional study of an outbreak of PD in Phuentsholing Bhutan, 2021.

The study was conducted in the Phuentsholing municipality, Chhukha District where the outbreak occurred. Of the 11 sub‐districts in Chhukha, Phuentsholing municipality is densely populated with >40% of the district population. The 2017 Population Housing Census of Bhutan recorded 27 658 individuals in Phuentsholing, excluding non‐Bhutanese and tourists.^9^ The municipality is the commercial hub of Bhutan with a diverse group of the transient population coming in from and travelling to all other districts.

### 3.2 Case definition

The case was defined in patients living in Phuentsholing between May and August 2021 with skin lesions, flat or linear with surrounding erythema and one of the following characteristics: lesions mimicking a burn with a crusty appearance or a vesicular lesion^3^, ^7^ with or without history or contact with a beetle.

### 3.3 Data collection

Based on the above case definition, data were collected from June to August 2021. After obtaining informed written consent, pictures of skin rash were taken and stored for use in this study. When patients sought medical attention at Phuentsholing hospital, a history of contact with the *Paederus* beetles was recorded. The majority of the patients did not know about the beetle until when shown the pictures of it, they agreed to have noticed or come in contact with the rove beetles. They were also informed to notify the investigators if they came across these beetles in their living places or surrounding. These pictures of the insects that we collected from the patients were sent to the Royal Entomological Society, United Kingdom via email that confirmed the genus *Paederus*, commonly known as rove beetles.

A questionnaire was used to collect socio‐demographic information (sex, age, among others), date and place of occurrence of dermatitis cases, residence type and clinical manifestations related to the lesions (anatomical location, symptoms, risk factors). Kissing lesions are characterized by the rash appearing near flexures which are mirror images of each other due to contact on limb lesions and, erythematovesicular is characterized by the presence of tiny vesicles in the background of an erythematous rash. The linear rash is those appearing longitudinal or linear with surrounding erythema and, the classic lesions with erythematovesicular and necrotic patterns. The patients who presented with a skin rash that have a doubtful diagnosis or alternative diagnoses and those who did not consent to this study were excluded.

We recorded all the medications that were prescribed during the clinical course of dermatitis. We did not undertake any intervention or introduce new treatments apart from the standard treatment prescribed by the local clinicians. A separate investigator was assigned for following up on the cases and measuring the outcome to prevent the investigators' evaluation from being influenced (intentionally or unintentionally) by their personal treatment preferences. Recovery was defined as the resolution of symptoms and a sign of healing. The follow‐up for the majority of patients was done at the hospital during review after 5 days of the initial hospital visit and then after every 3 days until recovery. Home visit was done for a small number of patients who could not visit the hospital after contacting them through the telephone.

### 3.4 Data entry and analysis

Data entry was done in Google sheet and the data analysis was carried out in STATA 13.1. Continuous variables are expressed as mean ± standard deviation and categorical data are expressed as a percentage. The descriptive analysis of the symptoms and signs, morphology and distribution of skin rash, the average duration of illness and recovery time are presented.

## 4 RESULTS

There were 102 suspected cases of beetle dermatitis where 12 did not consent to this study, and 9 accounted for the loss to follow‐up. The final sample consisted of 81 patients; there were 44 males (54.0%) and the mean age was 22 years (range: 1–51 years, SD: ±11.59 years). There were 80 (98.8%) Bhutanese and one was an Indian national. Those aged between 21 and 30 years (33, 40.7%) and school or college students (41, 50.6%) were the most affected age group. Notably, 61 (75.0%) patients had reported exposure to *Paederus* beetles before the onset of skin rash. These sociodemographic details of the patients with PD are shown in Table 1.

**TABLE 1:** Socio‐demographic details of patients with *Paederus* dermatitis in Phuentsholing, Bhutan, 2021.

Category	Number	Percent
Sex		
Male	44	54.3
Female	37	45.7
Age (years)		
≤10	18	22.2
11–20	13	16.1
21–30	33	40.7
31–40	11	13.6
≥41	6	7.4
Nationality		
Bhutanese	80	98.8
Indian	1	1.2
Reported exposure to *Paederus* beetles	61	75.0
Residence type		
Concrete houses	73	90.1
Temporary structures/camps	7	8.6
Traditional Bhutanese house	1	1.2
Level of education		
Cannot read and write	16	19.8
Primary school	17	21.0
Secondary school	27	33.3
College student	20	24.7
University degree	1	1.2
Occupation		
Student	41	50.6
Private/business	19	23.5
Homemaker	11	13.6
Minor (dependent)	7	8.6
Armed forces	2	2.5
Civil servant	1	1.2

**FIGURE 1:**
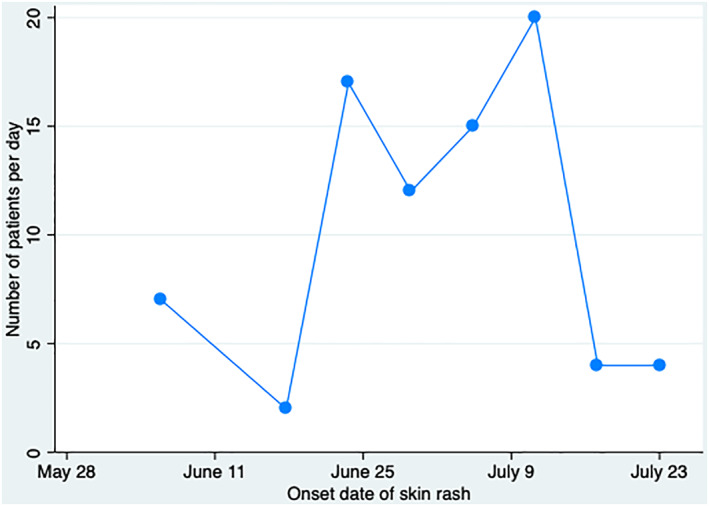
Time series of the outbreak of *Paederus* dermatitis in Phuentsholing, Bhutan, 2021.

**FIGURE 2:**
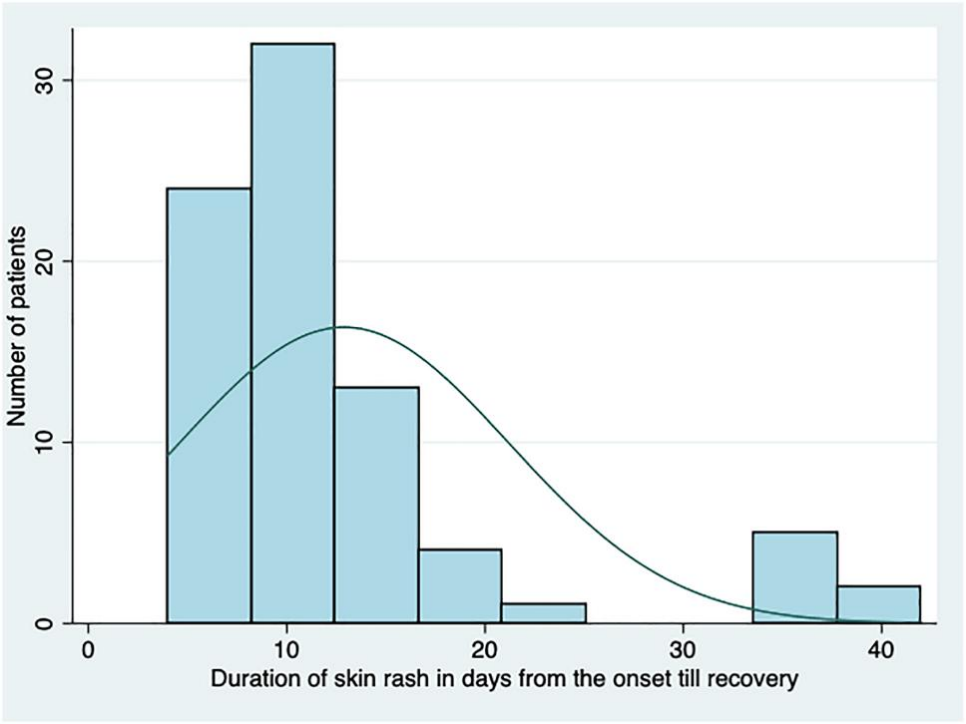
Distribution of skin rash duration from the onset till recovery of *Paederus* dermatitis patients in Phuentsholing, Bhutan, 2021.

The first case included in this study reported the onset of a rash on 3 June 2021. There was a surge in the number of cases in June and July as shown in Figure 1. The mean duration from the onset of symptoms till the recovery of skin rash was 13 days (SD ± 8.3 days) (Figure 2).

**FIGURE 3:**
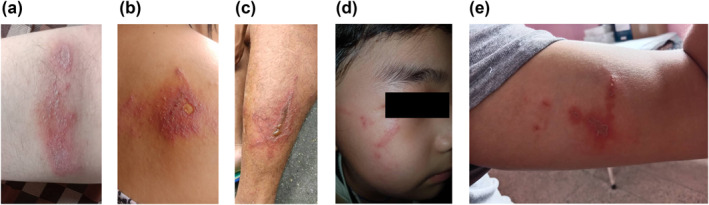
Skin lesions in patients with *Paederus* dermatitis. (a) Erythematovesicular rash on the left anterior forearm (a) and on the back with a ruptured blister at the centre (b). Linear dermatitis over the left lateral leg (c), the right side of the face (d) and on the right arm (e). Photo copyright: Authors.

**FIGURE 4:**
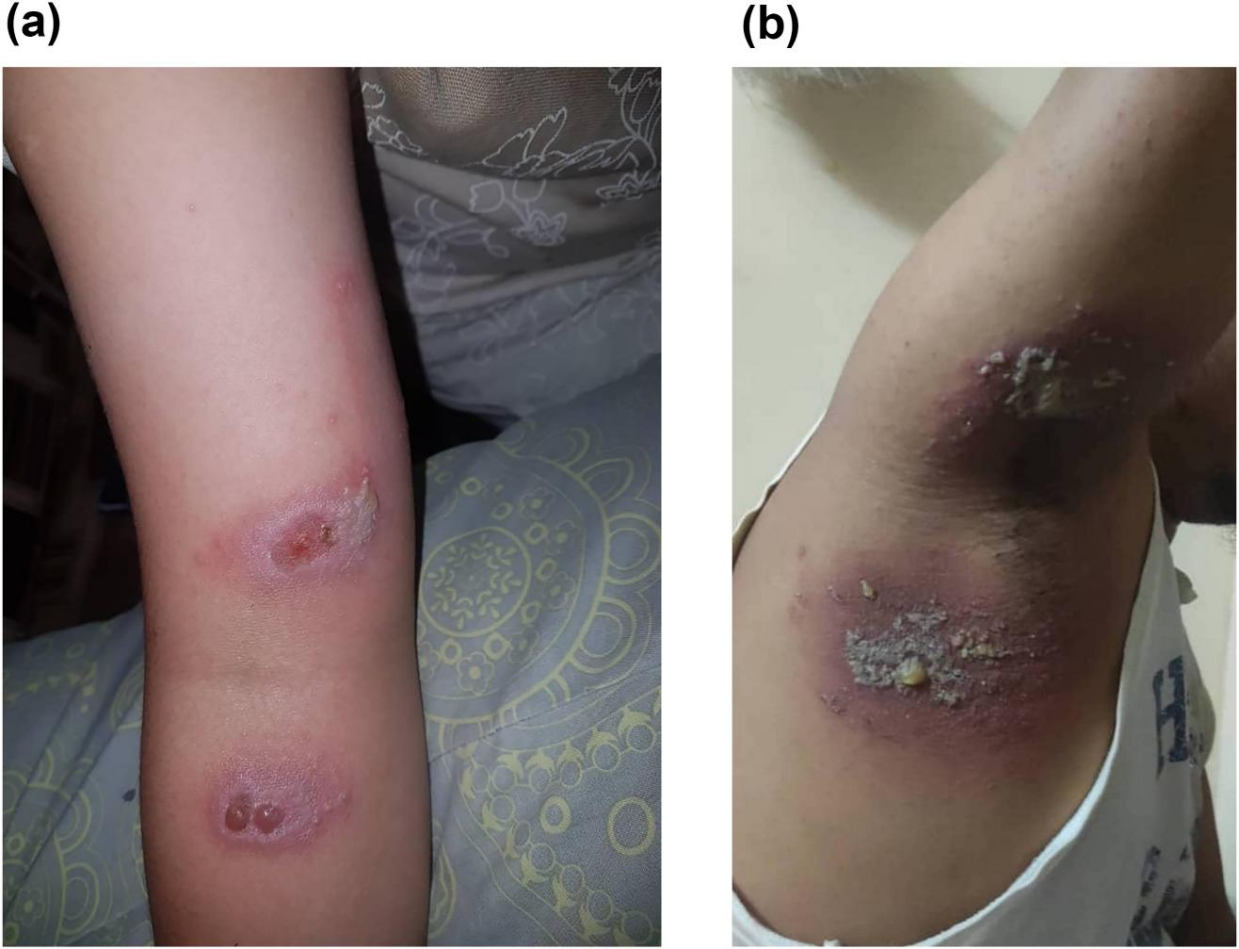
Kissing lesions of *Paederus* dermatitis appear on opposing surfaces that come into contact. (a) Erythematovesicular kissing rash with central ulceration which is partially healed over the right antecubital area. (b) Kissing lesions over the right axillary region with pustules.

The symptoms reported by the patients were pain (73, 90.0%), itching (67, 82.7%), redness (48, 59.3%) and tenderness (45, 55.6%). Fever, pus and watery discharge were reported by less than 8.0% of the patients (Table 2). Erythematovesicular was noted in 57 (70.4%) patients, characterized by the presence of tiny vesicles in the background of an erythematous rash (Figure 3(a, b)). Linear rash accounted for 44 (54.3%) and appeared longitudinal or linear with surrounding erythema (Figure 3(c–e)). The typical kissing lesions were noted in 23 (28.4%) patients (Figure 4(a, b)). Classic lesions with erythematovesicular and necrotic patterns were observed in 32 (39.5%) patients, annular in 38 (46.9%), papular in 26 (32.1%) and bullous lesions in 17 (21.0%) patients. The details of the description of the skin lesions are shown in Table 2. Common sites of lesions were the upper limb in 30 (37.0%), followed by a rash on at least two separate areas (upper limb, lower limb, body, face and head or neck) 28 (34.6%), and the head and neck 26 (32.1%) (Table 2).

**TABLE 2:** Symptoms and description of the skin lesions of *Paederus* dermatitis in Phuentsholing, Bhutan 2020.

Variables	Number	Percent
Symptoms		
Pain	73	90.1
Itching	67	82.7
Redness	48	59.3
Pain on touching	45	55.6
Pus discharge	4	4.9
Watery discharge	6	7.4
Fever	3	3.7
Lesion appearance		
Linear	44	54.3
Kissing	23	28.4
Classic^a^	32	39.5
Annular	38	46.9
Bullae/blister	17	21.0
Erythematovesicular	57	70.4
Papule	26	32.1
Resolving	3	3.7
Site of lesion		
Upper limb	30	37.0
Lower limb	25	30.9
Body (chest, abdomen and back)	23	28.4
Face	8	9.9
Head and neck (excluding face)	26	32.1
At least two separate areas^b^	28	34.6

^a^ Mixed erythematovesicular and necrotic pattern.

^b^ Lesions on at least two of the body parts listed above.

The majority (47, 58.0%) were prescribed local application of hydrocortisone ointment, followed by triamcinolone ointment 26 (32.0%), and the combination of ointment and oral prednisolone (Table 3). All the patients recruited for this study recovered with or without post‐inflammatory pigmentation.

**TABLE 3:** Treatments prescribed to patients with *Paederus* dermatitis, Phuentsholing, Bhutan, 2021.

Treatments prescribed	Route of administration	Number	Percent
Hydrocortisone	Topical	47	58.0
Triamcinolone	Topical	26	32.0
Triamcinolone and prednisolone	Topical and oral	4	5.0
Hydrocortisone and triamcinolone	Topical	1	1.3
Hydrocortisone and prednisolone	Topical and oral	3	3.7

### 4.1 Aetiological agent

**FIGURE 5:**
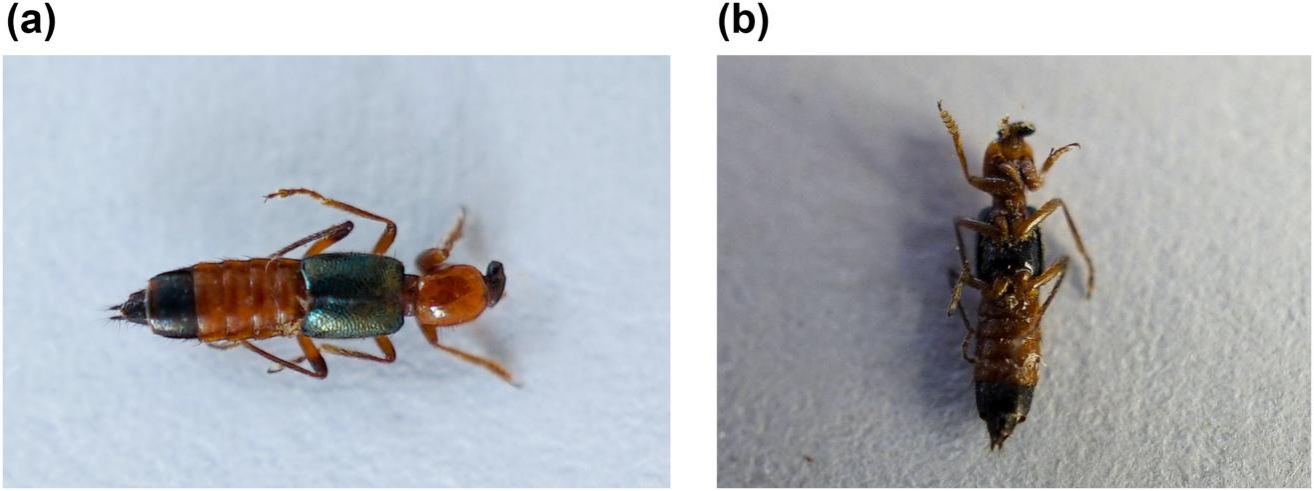
*Paederus* beetle spotted in homes in Phuentsholing, Bhutan in 2021. (a) Dorsal view, (b) ventral view. (Approximate dimension 8 × 1.5 mm).

Exposure to rove or *Paederus* beetles before the onset of skin rash was reported by 61 (75.3%) patients and the majority of them did not know that contact with these beetles would cause a skin rash. The beetle was identified as *Paederus* species by the online entomology information support group (Royal Entomological Society, United Kingdom) **(**Figure 5(a, b)).

## 5 DISCUSSION

The foothills of the eastern Himalayas across northeastern Indian states, Nepal and Bhutan have reported their hottest July summers, 2.5°C higher than the normal average temperatures of 25.6°C in 2021 and 2022.^10^ This corresponded with the sighting of *Paederus* beetles and an outbreak of PD in this region. While numerous outbreaks of blister beetle dermatitis were reported worldwide,^4^, ^11^, ^12^ some of them have been linked to an increased population of beetles. Beetles are most active during the rainy season, after unusually wet weather patterns, and during hot and humid weather.^13^ Humid, wet conditions prevent desiccation of the beetle during flight and movement thereby encouraging higher levels of activity and wider geographic ranges of dispersal.^14^, ^15^

The outbreak of PD in the Phuentsholing subdistrict in the years 2021 and 2022 led to the spread of fear and panic among populations living in the subtropical belt of Bhutan. Many patients have had epidemiological linkage with the increased beetle population in their residences or surroundings. In addition, there were many other reports of *Paederus* beetles associated with outbreaks of dermatitis in many places in the northeastern Indian states of West Bengal, Sikkim and Assam and along many districts in Nepal. In recent years, many countries across the world including Central Africa, Uganda, Sierra Leone, Argentina, Brazil, France, Sri Lanka and India have reported PD.^6^, ^16^

The predominant symptoms reported were pain, itching, redness and tenderness in overexposed parts of the body, similar to findings reported in other countries.^17^ The majority of lesions were linear erythematous and erythemato‐vesicular with a ‘burnt’ or crusty appearance and a grey necrotic centre.^7^, ^18^ In mild cases, the erythema lasts for a couple of days, whereas in moderate cases, the erythema evolves into vesicles and bullae over a few days and is followed by desquamation. Scarring usually does not occur. The lesions are characteristically linear due to smearing the crushed insect across the skin.^19^ Severe cases are reported with more extensive blistering and may demonstrate additional symptoms, such as fever, neuralgia, arthralgia and vomiting.^20^, ^21^ In the absence of a history of exposure to beetles, there are other differential diagnoses to consider including allergic dermatitis, arthropod bites, herpes zoster, herpes simplex, bullous impetigo, psoriasis and fungal infections.^22^

The mean duration of skin rash from the onset until recovery was 13 days. Reports from India and Italy show almost similar duration to recovery with 12–15 days,^21^, ^23^ whereas another study from Sierra Leone suggested that healing time ranged from 14 to 28 days and lesions in all the patients healed with residual dyschromia.^20^ Although the condition is self‐limiting, the symptoms can be distressing for the patients and may affect their daily activities. Complications such as superadded infections are relatively rare.

In our study, steroid treatment was prescribed with an understanding that PD is one form of contact dermatitis.^24^ Though symptoms and signs can resolve spontaneously, wet compresses, antihistamines and lotions are recommended to alleviate symptoms.^13^, ^25^

PD, though self‐limiting, is likely to cause a major public health concern given the background of unchecked warming of the climate and an increasing population of rove beetles. This outbreak led to fear and panic among residents in the sub‐tropical region and increased healthcare utilization, which is a burden, especially in Bhutan where it is provided free of cost. This also led to temporary disruption of education and work where outbreaks were reported in schools and hostels in northeastern parts of India.^15^ Such outbreaks necessitate appropriate preventive measures. It is advised to minimize artificial light sources where these beetles are attracted. During the contact of the beetle with skin, it is advised to avoid crushing the beetle against the skin (blow it off instead of crushing it), close the doors and windows at night during the season of an outbreak, washing the affected body area with soap and clean water, and the application of cold wet compresses.^24^, ^25^, ^26^

So far, very little is known regarding the application of insecticide to control *the Paederus* beetle population. Therefore, the health authorities must place a robust surveillance system to forecast massive outbreaks. People living in tropical regions must be given education and awareness about blister beetle dermatitis and the need to adopt preventive measures.

Climate change has particular consequences on the emergence or re‐emergence of infectious diseases, and the impact can transcend beyond national boundaries.^27^ This outbreak in the foothills of the eastern Himalayas is not an isolated phenomenon. The region is witnessing a northward shift in disease agents which, to make matter worse, are further spread by the increase in travelling within and outside the region. For example, travelling to places with increased beetle populations has resulted in contracting PD among the returning travellers in Italy from Zanzibar island,^22^ and in Virginia among travellers who returned from Sierra Leone.^28^ This calls for action from health systems across the world to develop mechanisms to detect and monitor the emergence of new disease agents and mechanisms to respond to such outbreaks and importantly to limit their spread and prevent future outbreaks.

## 6 CONCLUSION

PD is characterized by a painful, itchy and blistering rash that is predominantly erythematovesicular or linear and is due to exposure to *Paederus* or rove beetles. The outbreak in Phuentsholing occurred in the background of an unusually hot summer with temperatures higher than the historical records. Emerging infectious and vector‐borne diseases will continue to challenge health systems as the climate remains unchecked.

## 7 LIMITATIONS OF THE STUDY

The size of the sample might not represent everyone affected by the outbreak of PD in Phuentsholing due to several reasons: some patients with milder symptoms might not have come to the hospital; some of the affected patients with shorter duration of skin rash or with relatively small lesions may have missed. In addition, there might have been selection bias as this was a hospital‐based study.

## CONFLICT OF INTEREST STATEMENT

The authors declare that they have no competing interests.

## AUTHOR CONTRIBUTIONS

**Kinley Gyeltshen**: Conceptualization (equal); Formal analysis (equal); Investigation (lead); Methodology (equal); Project administration (lead); Writing – Original draft (lead); Writing – review and editing (equal). **Ngawang Sangye**: Conceptualization (equal); Formal analysis (equal); Investigation (equal); Methodology (equal). **Kunzang C. Tenzin**: Conceptualization (equal); Formal analysis (equal); Investigation (equal); Methodology (equal); Writing – Original draft (supporting). **Thinley Dorji**: Conceptualization (equal); Formal analysis (equal); Methodology (equal); Writing – review and editing (lead).

## ETHICS STATEMENT

The study was conducted according to the principles of the Declaration of Helsinki and was approved by the Research Ethics Board of Health, Ministry of Health, Bhutan (Ref. No. REBH/Approval/2021/129) dated 2 November 2021. Prior permission was granted by the Policy and Planning Division, Ministry of Health and by the administrator of Phuentsholing General Hospital to conduct this study. Informed written consent was taken from the patients to use the photographs of their skin rash. The consent form was approved by the Research Ethics Board of Health, Bhutan.

## Data Availability

Data is available at request from the Ministry of Health, Bhutan.
